# Naturally occurring layered mineral franckeite with anisotropic Raman scattering and third-harmonic generation responses

**DOI:** 10.1038/s41598-021-88143-5

**Published:** 2021-04-19

**Authors:** Ravi P. N. Tripathi, Jie Gao, Xiaodong Yang

**Affiliations:** Department of Mechanical and Aerospace Engineering, Missouri University of Science and Technology, Rolla, MO 65409 USA

**Keywords:** Two-dimensional materials, Nonlinear optics

## Abstract

Vertically stacked van der Waals (vdW) heterostructures have introduced a unique way to engineer optical and electronic responses in multifunctional photonic and quantum devices. However, the technical challenges associated with the artificially fabricated vertical heterostructures have emerged as a bottleneck to restrict their proficient utilization, which emphasizes the necessity of exploring naturally occurring vdW heterostructures. As one type of naturally occurring vdW heterostructures, franckeite has recently attracted significant interest in optoelectronic applications, but the understanding of light–matter interactions in such layered mineral is still very limited especially in the nonlinear optical regime. Herein, the anisotropic Raman scattering and third-harmonic generation (THG) from mechanically exfoliated franckeite thin flakes are investigated. The observed highly anisotropic Raman modes and THG emission patterns originate from the low-symmetry crystal structure of franckeite induced by the lattice incommensurability between two constituent stacked layers. The thickness-dependent anisotropic THG response is further analyzed to retrieve the third-order nonlinear susceptibility for franckeite crystal. The results discussed herein not only provide new insights in engineering the nonlinear light–matter interactions in natural vdW heterostructures, but also develop a testbed for designing future miniaturized quantum photonics devices and circuits based on such heterostructures.

## Introduction

In recent years, vertically stacked van der Waals (vdW) heterostructures have facilitated an unprecedented opportunity to tailor the optical and electronic properties of two-dimensional (2D) materials at the atomic scale^1–3^, which reside at the heart of many advanced photonic and quantum devices such as light-emitting diodes^4^, field-effect transistors^5^, photodetectors^6^, ultrafast pulsed lasers^7,8^, optical modulators^9,10^, atomically-thin holograms^11^, spintronic devices^12^, nonvolatile memory devices^13^, and superconducting circuits^14^. Vertical heterostructures are usually constructed by manually stacking the dissimilar 2D material layers on top of each other, in order to obtain unconventional physical properties which are otherwise inaccessible with the individual layer^1–3,15,16^. However, the artificially fabricated vertical heterostructures suffer from several technical challenges including the poor crystalline alignment control, the potential polymer contaminations, and the unwanted interlayer adsorbates^15,17^. Moreover, the sheet-by-sheet construction of vertical heterostructures has been demonstrated mainly for some specific types of 2D materials such as transition metal dichalcogenides, graphene and hexagonal boron nitride (hBN)^2,11,15,18–20^. Therefore, it is highly imperative to explore alternative approaches for obtaining vertical heterostructures in order to enable the full-fledged utilization of vdW heterostructures in future applications. One promising alternative approach is the mechanical exfoliation of natural vdW heterostructures.

In this regard, as one type of naturally occurring vertical vdW heterostructures, franckeite has recently attained significant interest for advanced optoelectronic applications^21–25^. Sulfosalt minerals are complex mixed-metal chalcogenides with the general formula of A_m_B_n_S_p_, where A represents metals like copper, lead, silver or iron, B is semimetals such as arsenic, antimony, bismuth or metals like tin, and S is sulfur^26^. Among all these sulfosalt minerals, franckeite (Pb_5_Sn_2_FeSb_2_S_14_) has gained substantial attention due to its promising features and easy exfoliation. Franckeite consists of alternating pseudo-hexagonal (H) SnS_2_-like layer and pseudo-tetragonal (Q) PbS-like layer, forming natural vdW heterostructures. So far, field-effect devices and photodetectors have been demonstrated with mechanically exfoliated franckeite flakes^21,22,27,28^, showing that franckeite is a p-type semiconductor with a narrow band gap around 0.65 eV. The shear-exfoliated franckeite nanosheets have been shown as electrocatalysts for energy related applications to improve hydrogen evolution performance^29^. The optical properties of layered franckeite have also been studied including the refractive index in the visible spectrum determined by a quantitative analysis of the optical contrast spectra, as well as the third-order nonlinear optical properties characterized by Z-scan and spatial self-phase modulation techniques^23,30^. In addition, it has been recently shown that franckeite exhibits the spontaneous symmetry breakdown due to the PbS-like and SnS_2_-like lattice incommensurability, which results in anisotropic electrical, vibrational and optical responses^24^. Although many research efforts have been devoted to studying the nonlinear optical properties of 2D layered vdW materials^31^, there is no comprehensive study presented on the anisotropic nonlinear optical responses in the incommensurate heterostructures of franckeite yet.

Motivated by this, herein we demonstrate the anisotropic Raman scattering response as well as the anisotropic third-harmonic generation (THG) response from mechanically exfoliated franckeite thin flakes. The angle-resolved polarized Raman spectroscopy and the polarization-dependent THG emission are utilized in probing the anisotropic natural vdW heterostructures and identifying the rippling direction of franckeite crystals. The Raman modes in franckeite crystal are found highly anisotropic under both parallel and perpendicular polarization configurations. It is observed that the THG emission pattern is also highly anisotropic with respect to the incident linearly polarized pump beam, arising from the symmetry-broken periodic ripples in the crystal structure of franckeite induced by the lattice incommensurability and in-plane spatial modulation of vdW interaction between the consecutively stacked PbS-like and SnS_2_-like layers. The measured anisotropic THG response is further corroborated with the theoretical nonlinear susceptibility model, which enables the determination of the relative magnitudes of the third-order nonlinear susceptibility tensor elements for franckeite crystal. The effect of the franckeite flake thickness on the THG emission power is further explored to determine the value of the third-order nonlinear susceptibility. We anticipate that these results will provide new insights into the comprehensive understanding of light–matter interactions in natural vdW heterostructures and in realizing advanced quantum photonics devices for future applications in integrated photonic circuits, polarization-based entangled photon generation, encrypted optical signal processing, and quantum information science.

## Results

### Anisotropic Raman response of franckeite thin flakes and identification of crystal rippling direction

**Figure 1:**
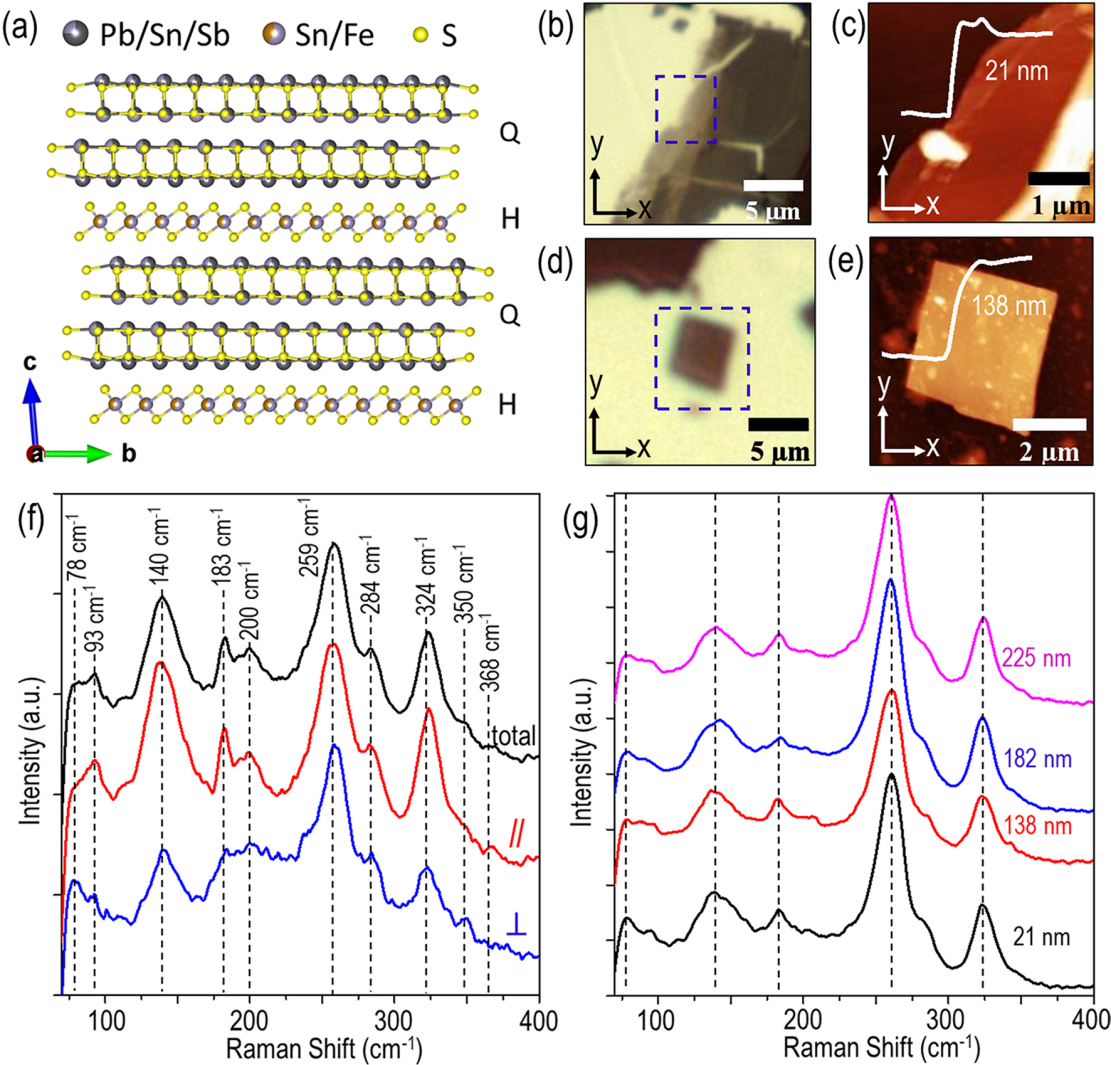
(**a**) Schematic side view of franckeite crystal structure. (**b**,**d**) Transmission microscope images of two franckeite thin flakes exfoliated on a glass substrate. Blue dashed box marks the experimentally probed region, whereas *x*-axis and *y*-axis signify the crystal’s *b*-axis and *a*-axis, respectively. (**c**,**e**) AFM images of the marked regions in the transmission microscope images. The line profiles in the insets signify the thickness of the probed regions. (**f**) Acquired Raman spectra from a typical exfoliated franckeite flake with 138 nm thickness in the total (black curve), parallel (red curve) and perpendicular (blue curve) polarization configurations. The corresponding Raman peaks are indicated by the black dashed lines. (**g**) Comparison of Raman spectra under the parallel configuration as a function of the franckeite flake thickness.

Franckeite crystal consists of the repetitive stacking of two vdW layers along the *c*-axis, one is the PbS-like layer (Q-layer) and the other is the SnS_2_-like layer (H-layer), interacting together with vdW forces^21,22^. Figure 1a illustrates the simplified version of atomic layer arrangement (Q-layer and H-layer) in a typical franckeite crystal. The Q-layer consists of four atomic layers of sulfide compound with the general molecular formulation as MX, where M = Pb^2+^, Sn^2+^ or Sb^3+^ and X = S with the unit cell parameters *a* = 5.84 Å, *b* = 5.90 Å, and *c* = 17.3 Å^32^. The H-layer is composed of the octahedrons of sulfide compound with the general molecular formulation of MX_2_, where M = Sn^4+^ or Fe^2+^ and X = S with the unit cell parameters *a* = 3.68 Å, *b* = 6.32 Å, and *c* = 17.3 Å^32^. Franckeite has a triclinic crystal structure belonging to the $P\overline{1 }$ space group. The lattice incommensurability of two consecutively stacked PbS-like and SnS_2_-like layers introduces a strain-induced structural deformation, which develops symmetry-broken periodic ripples with a period of around 4.7 nm along the *b*-axis^24,32^. Such one-dimensional rippling of the heterostructure will give rise to the in-plane anisotropic physical properties for franckeite crystal in the *a–b* plane. Franckeite thin flakes are prepared by the typical mechanical exfoliation process using Nitto tape (SPV 224) from bulk natural franckeite mineral (from San José mine, Oruro, Bolivia) and transfer to a glass substrate. Figure 1b,d show the transmission microscope image of two exfoliated franckeite flakes, where the variations in optical contrast indicate different thicknesses of franckeite flakes. The experimentally interested regions are marked by a blue dashed box in Fig. 1b,d. Atomic force microscopy (AFM) is used to scan the interested regions to check the surface smoothness and flake thickness. The AFM scanned profiles are shown in Fig. 1c,e, where the flake thickness is found approximately 21 nm and 138 nm, respectively.

The crystal axes of franckeite crystal can be identified from the Raman spectroscopy. First, the Raman spectra from the 138 nm-thick franckeite flake are characterized and analyzed. Figure 1f shows the measured Raman spectra with a 632.8 nm He–Ne laser as the excitation source in three different polarization analyzer configurations. The total Raman spectrum is measured without the analyzer after the sample. In the parallel or perpendicular configuration, the analyzer direction is set to be parallel or perpendicular to the linear polarization direction of the excitation beam. The Raman spectrum of franckeite exhibits several distinct Raman peaks within 70–400 cm^−1^, which are located at 78, 93, 140, 183, 200, 258, 284, 324, 350 and 368 cm^−1^. The Raman modes of franckeite can be assigned according to the Raman spectra of the constituent components, including galena PbS^33^, herzenbergite SnS^34,35^, berndtite SnS_2_^36^, pyrite FeS_2_^37^, and stibnite Sb_2_S_3_^38,39^. The 78 cm^−1^ peak is assigned as the A_g_ mode of Sb_2_S_3_ (73 cm^−1^). The peak at 93 cm^−1^ is attributed to the A_g_ mode of SnS (95 cm^−1^). The 140 cm^−1^ peak corresponds to a combination of phonon modes of both SnS_2_ lattices (the second-order effects at 140 cm^−1^) and PbS lattices (transverse acoustic and optical phonon modes at 154 cm^−1^). The 183 cm^−1^ peak is related to a combination of the A_g_ mode of SnS (192 cm^−1^) and the A_g_ mode of Sb_2_S_3_ (191 cm^−1^). The 200 cm^−1^ peak is attributed to a combination of the E_g_ mode of SnS_2_ (202 cm^−1^) and the longitudinal optical phonon modes of PbS lattices (204 cm^−1^). The 258 cm^−1^ peak represents the B_1g_/B_3g_ mode of Sb_2_S_3_ (238 cm^−1^), while the 284 cm^−1^ peak belongs to the A_g_ mode of Sb_2_S_3_ (283 cm^−1^). The peak at 324 cm^−1^ is assigned as a combination of the A_g_ mode of Sb_2_S_3_ (312 cm^−1^) and the A_1g_ mode of SnS_2_ (315 cm^−1^). Finally, two weak Raman peaks at 350 cm^−1^ and 368 cm^−1^ belong to the E_g_ mode (337 cm^−1^) and the A_g_ mode (372 cm^−1^) of FeS_2_, respectively. In addition, it is noticed that the majority of modes (93, 140, 183, 200, 259, 284 and 324 cm^−1^) appear in both the parallel and perpendicular configurations, and only few modes (78, 350 and 368 cm^−1^) can be observed in either configuration. The effect of the flake thickness variation on the Raman shift is also probed. Figure 1g shows the measured Raman spectra for the franckeite flakes with thicknesses of 21, 138, 182 and 225 nm under the parallel configuration. No significant change in the Raman shift is observed, which is expected for franckeite crystal due to the incommensurate stacking of the Q-layer and H-layer^22^. However, the intensity variations in Raman modes are observed as the flake thickness changes, which can be attributed to the optical interference and the associated optical field enhancement within the layered materials^22^.

**Figure 2:**
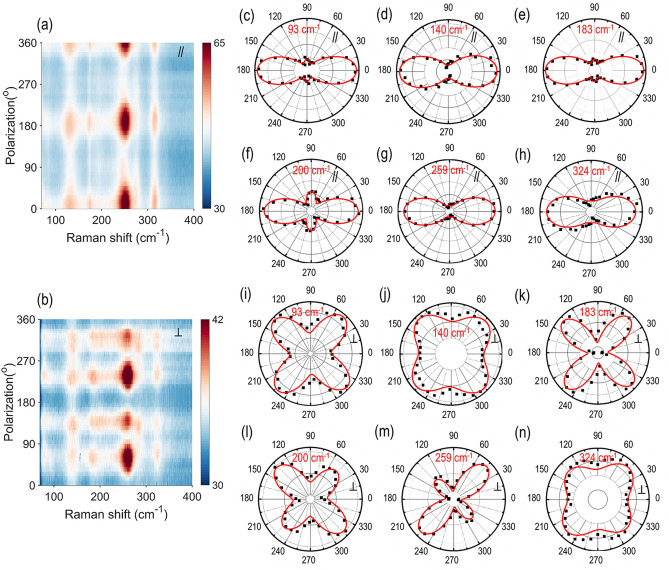
(**a**,**b**) Contour maps of angle-resolved polarized Raman spectra for the 138 nm-thick franckeite flake in the parallel (//) and perpendicular ($\perp$) configurations. (**c**–**h**) Polar plots of the Raman A_g_ modes at 93, 140, 183, 200, 259 and 324 cm^−1^ in the parallel configuration. (**i**–**n**) Polar plots of the Raman A_g_ modes in the perpendicular configuration. Black squares are the experimental data and red solid curves are the corresponding theoretical fits.

Next, the angle-resolved polarized Raman spectroscopy is performed to identify the in-plane rippling direction along the *b*-axis of franckeite crystal. Figure 2a,b show the Raman intensity contour maps depending on the linear polarization angle of the excitation beam for the 138 nm-thick franckeite flake in the parallel and perpendicular configurations, which clearly shows that the Raman mode intensities are highly polarization sensitive. The periodic variations of Raman mode intensities with respect to the linear polarization angle of the excitation beam are observed. In general, the Raman mode intensity can be expressed as

1 $$I \propto {\left|{e}_{i}\cdot R{\cdot e}_{s} \right|}^{2}$$

where ${e}_{i}$ and ${e}_{s}$ denote the unit polarization vectors for the incident and scattered light, and $R$ is the Raman tensor for the probed crystal. In accordance with the experimental configuration, ${e}_{i}$ and ${e}_{s}$ are in the *x*–*y* plane and there is no contribution from the *z*-axis. Hence, the unit polarization vectors can be written as ${e}_{i}=(cos\theta , sin\theta , 0)$, while ${e}_{s}=(cos\theta , sin\theta , 0)$ and $(-sin\theta , cos\theta , 0)$ for the parallel and perpendicular polarization configurations, respectively. The Raman tensor of A_g_ modes for the triclinic franckeite crystal can be written as

2 $$R= \left[\begin{array}{cc}u& v\\ v& w\end{array}\right]$$

where *u*, *v* and *w* are the Raman tensor components. Thus, the Raman intensity of the A_g_ modes in the parallel and perpendicular configurations can be written as follows^40^

3 $$I_{{A_{g} }}^{{//}} \propto \left( {ucos^{2} \theta + 2vsin\theta cos\theta + wsin^{2} \theta } \right)^{2}$$

4 $${I}_{{A}_{g}}^{\perp }\propto {(v[{cos}^{2}\theta - {sin}^{2}\theta ]+[w-u]sin\theta cos\theta )}^{2}$$

where // and $\perp$ denote the parallel and perpendicular configurations. Figure 2c–h show the polar plots of the Raman A_g_ modes at 93, 140, 183, 200, 259 and 324 cm^−1^ measured under the parallel configuration, whereas Fig. 2i–n give the polar plots for the respective Raman modes under the perpendicular configuration. It is found that the experimental observations (black squares) agree well with the theoretical fits (red solid curves). Notably, under the parallel configuration the Raman intensities of A_g_ modes exhibit highly anisotropic two-lobe patterns with a period of 180°, where the intensity reaches the maximum at 0° and 180°. On the other hand, under the perpendicular configuration the Raman intensity patterns show the anisotropic four-lobe patterns with the maximum and the second maximum intensities located around either 45° and 225° or 135° and 315°. Moreover, the angle-resolved Raman mode analysis can be used to identify the crystal axis^24,41–43^. Based on the current observations, it is confirmed that *x*-axis (0°) and *y*-axis (90°) are identified as the rippling direction (along the *b*-axis) and its perpendicular direction (along the *a*-axis), respectively.

### Anisotropic THG response in franckeite thin flakes and determination of ${{\varvec{\chi}}}^{(3)}$

**Figure 3:**
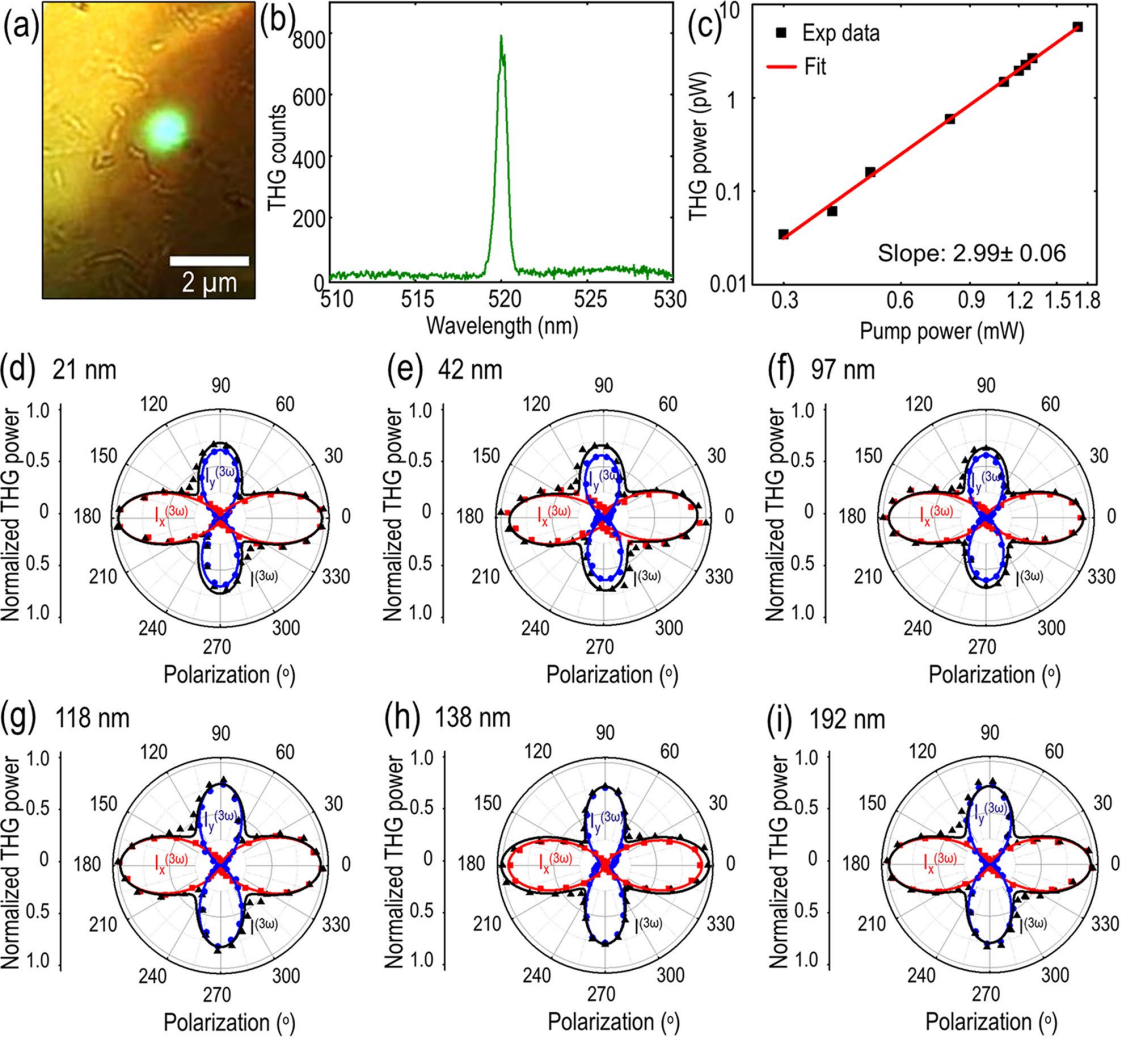
(**a**) Transmission microscope image with THG emission from the 21 nm-thick franckeite flake. (**b**) Measured THG emission spectrum showing the peak emission wavelength at 520 nm which is exactly one-third of the excitation wavelength at 1560 nm. (**c**) Log-scale plot of the THG emission power as a function of the pump power. (**d**–**i**) Angular dependence of the THG emission power on the incident linear polarization angle for six franckeite flakes with the thicknesses of 21, 42, 97, 118, 138 and 192 nm. Black triangles, red squares and blue circles correspond to the measured total, *x*-component and *y*-component of the THG emission power, ${I}^{(3\omega )}$, ${I}_{x}^{(3\omega )}$ and ${I}_{y}^{(3\omega )}$, respectively. The corresponding theoretical fittings are plotted as solid curves.

It has been recently demonstrated that franckeite crystal exhibits strong linear dichroism in visible light absorption due to its structural deformation^24^, but the anisotropic nonlinear optical response and the corresponding third-order nonlinear susceptibility tensor in these heterostructures are still unknown and need to be investigated. Figure 3a shows the transmission microscope image with the green THG emission from the 21 nm-thick franckeite flake excited by a 1560 nm pump laser with the spot size of 1.5 µm. The THG emission spectrum is plotted in Fig. 3b, showing the peak emission wavelength at 520 nm which is exactly one-third of excitation wavelength. To further confirm the THG emission, Fig. 3c shows the log-scale plot of the THG emission power as a function of the pump power, where the cubic power law fitting endorses the THG emission process.

Next, the in-plane anisotropic THG emission from franckeite crystal is investigated by measuring the THG intensity dependence on the input linear polarization of the pump beam. The desired incidence linear polarization angle of the pump beam with respect to the rippling direction of the crystal is obtained by using a linear polarizer and a rotating half-wave plate placed in the excitation path before the sample. Also, a linear polarization analyzer is fixed at either 0° or 90° after the sample for measuring the *x* and *y* components of THG emission. Figure 3d–i plot the angular dependence of the THG emission power on the incident linear polarization angle with respect to the *x*-axis (rippling direction) for six franckeite flakes with the thicknesses of 21, 42, 97, 118, 138 and 192 nm, showing highly anisotropic four-lobe THG emission patterns. The black triangles represent the measured total THG emission power, while the red squares and blue circles correspond to the *x* and *y* components of the THG power. The maximum THG power is observed when the incident linear polarization angle is 0° along the *x*-axis (rippling direction) and the second maximum THG power occurs at 90° along the *y*-axis. The observed anisotropic THG emission from franckeite crystal originates from the symmetry breaking and generation of periodic ripples in franckeite lattice, which is induced by the lattice incommensurability and in-plane spatial modulation of vdW interaction between the consecutively stacked Q- and H-layers. The origin of the spatially modulated strain and generation of ripples in franckeite lattice has been recently presented^24^. The vdW adhesion energy between the consecutive Q- and H-layers is strongly dependent on the local atomic arrangements, which in turn depends on the inhomogeneous elastic deformation in the crystal lattice. As the crystal lattice minimizes its total energy (elastic plus adhesive), strong in-plane modulation of van der Waals forces and the out of plane ripples are generated in the crystal structure. As a result, the local density of states for light–matter interaction vary along both the crystal axes, leading to the anisotropic THG emission. To obtain further insights into the anisotropic THG response, the experimental observations are corroborated with the third-order nonlinear susceptibility model. As per the experimental configuration, the franckeite flake is illuminated with the linearly polarized pump beam with fundamental frequency *ω*. Therefore, the incident electric field can be expressed as $\overrightarrow{E}=|E|\widehat{p}$, where $\widehat{p}$ can be further connected with the rippling direction as $\widehat{p }=\widehat{x}cos\theta +\widehat{y}sin\theta$ with $\theta$ as the linear polarization angle relative to the rippling direction (*x*-axis). By considering the triclinic crystal structure of franckeite, the contracted form of its third-order nonlinear susceptibility tensor ${\chi }^{(3)}$ can be written as^44,45^

5 $${\chi }^{(3) }=\left[\begin{array}{ccc}{\chi }_{11}& {\chi }_{12}& {\chi }_{13}\\ {\chi }_{21}& {\chi }_{22}& {\chi }_{23}\\ {\chi }_{31}& {\chi }_{32}& {\chi }_{33}\end{array} \begin{array}{ccc}{\chi }_{14}& {\chi }_{15}& {\chi }_{16}\\ {\chi }_{24}& {\chi }_{25}& {\chi }_{26}\\ {\chi }_{34}& {\chi }_{35}& {\chi }_{36}\end{array} \begin{array}{ccc}{\chi }_{17}& {\chi }_{18}& {\chi }_{19}\\ {\chi }_{27}& {\chi }_{28}& {\chi }_{29}\\ {\chi }_{37}& {\chi }_{38}& {\chi }_{39}\end{array} \begin{array}{c}{\chi }_{10}\\ {\chi }_{20}\\ {\chi }_{30}\end{array} \right]$$

where the first term in subscript 1, 2, and 3 denotes *x*, *y* and *z* respectively and the second subscript refers to the combination of three components as

6 $$\begin{array}{ccc}xxx& yyy& zzz\\ 1& 2& 3\end{array} \begin{array}{ccc}yzz& yyz& xzz\\ 4& 5& 6\end{array} \begin{array}{ccc}xxz& xyy& xxy\\ 7& 8& 9\end{array} \begin{array}{c}xyz\\ 0\end{array}$$

As the polarization of the excitation field always remains in the *x–y* plane in the experimental configuration, no contribution from the components of ${\chi }^{\left(3\right)}$ containing *z* terms will be observed in the THG emission. Thus, only eight non-zero elements (${\chi }_{11}$, ${\chi }_{12}, {\chi }_{18}$, ${\chi }_{19}$, ${\chi }_{21}$, ${\chi }_{22}$, ${\chi }_{28}$ and ${\chi }_{29}$) of ${\chi }^{\left(3\right)}$ will contribute to the THG emission process. Then the electric field components of THG emission and the THG intensity can be expressed as follows

7 $${E}^{(3\omega )}= \left[\begin{array}{c}{E}_{x}^{(3\omega )}\\ {E}_{y}^{(3\omega )}\\ {E}_{z}^{(3\omega )}\end{array}\right] \propto {\varepsilon }_{0}{E}^{3}\left[\begin{array}{c}{\chi }_{11} {cos}^{3}\theta +{\chi }_{12} {sin}^{3}\theta + 3{\chi }_{18} cos\theta {sin}^{2}\theta + 3{\chi }_{19} sin\theta {cos}^{2}\theta \\ {\chi }_{21} {cos}^{3}\theta + {\chi }_{22} {sin}^{3}\theta +3{\chi }_{28} cos\theta {sin}^{2}\theta + 3{\chi }_{29} sin\theta {cos}^{2}\theta \\ 0\end{array}\right]$$

8 $${I}_{x}^{(3\omega ) }\propto {\left({\chi }_{11} {cos}^{3}\theta +{\chi }_{12} {sin}^{3}\theta + 3{\chi }_{18} cos\theta {sin}^{2}\theta + 3{\chi }_{19} sin\theta {cos}^{2}\theta \right)}^{2}$$

9 $${I}_{y}^{(3\omega ) }\propto {\left({\chi }_{21} {cos}^{3}\theta + {\chi }_{22} {sin}^{3}\theta +3{\chi }_{28} cos\theta {sin}^{2}\theta + 3{\chi }_{29} sin\theta {cos}^{2}\theta \right)}^{2}$$

The obtained THG intensity expressions along *x-* and *y*-axis are further used to fit the experimentally measured values. The theoretical fitting curves are plotted as the solid curves in the respective colors in Fig. 3d–i, showing a good agreement with the measurements.

**Figure 4:**
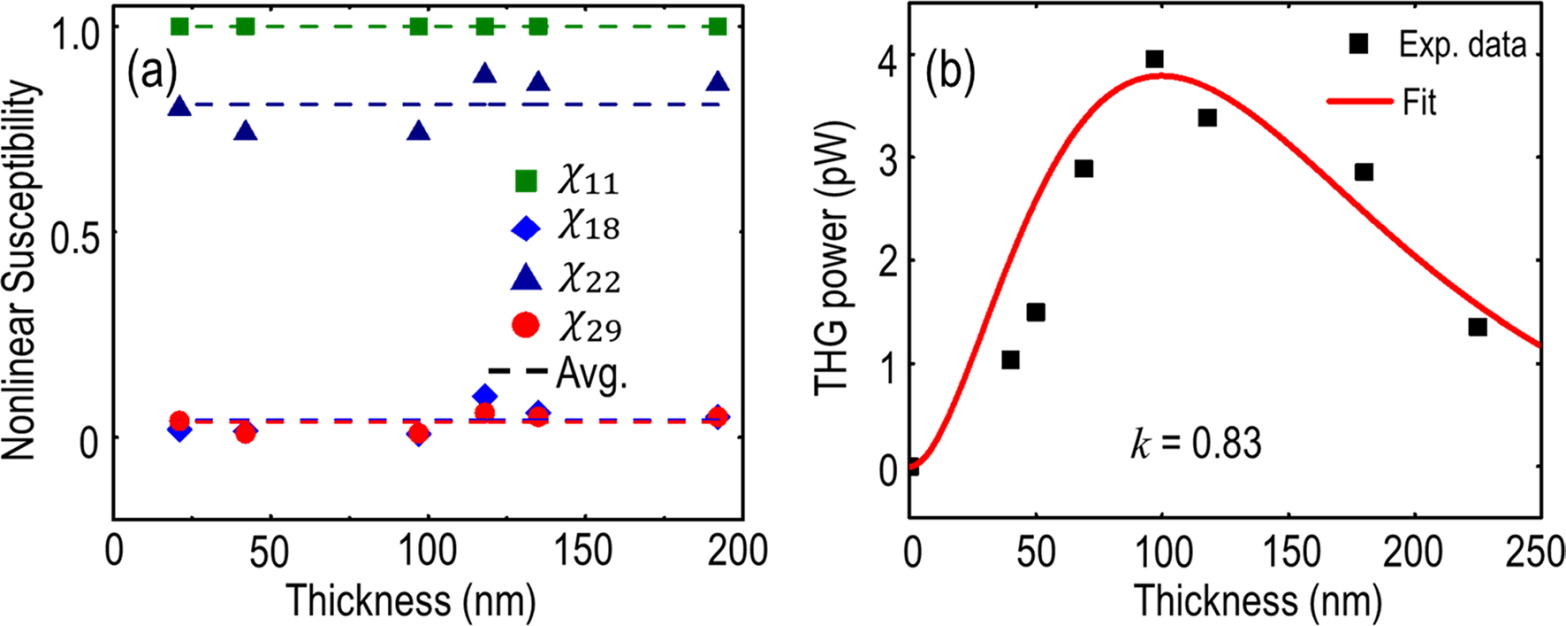
(**a**) Extracted relative magnitudes of ${\chi }_{11}$,${\chi }_{18}$, ${\chi }_{22}$ and ${\chi }_{29}$ with respect to the flake thickness, plotted as green squares, blue rectangles, royal blue upper triangles and red circles, respectively. The average values are marked as dashed lines with the associated colors. (**b**) Dependence of the THG emission power on the franckeite flake thickness. Experimental data and theoretical fitting are plotted with black squares and red solid curve, respectively.

Moreover, the relative magnitudes of ${\chi }^{(3)}$ tensor elements for franckeite crystal can be retrieved based on Eqs. (8) and (9). Figure 4a plots the obtained relative magnitudes of ${\chi }_{11}$,${\chi }_{18}$, ${\chi }_{22}$ and ${\chi }_{29}$ with respect to the flake thickness, indicating that the values of ${\chi }^{(3)}$ tensor elements remain almost unchanged for franckeite flakes with different thicknesses. The average relative magnitudes of ${\chi }^{(3)}$ tensor elements are ${\chi }_{11}: {\chi }_{12}:{\chi }_{18}:{\chi }_{19}: {{\chi }_{21}:\chi }_{22}:{{\chi }_{28}:\chi }_{29}=1:0.012:0.043:0.015:0.017:0.815:0.009:0.037$ which manifests the intrinsic anisotropic nonlinear optical properties of franckeite crystal. In addition, the ratio of ${{|\chi }_{11}|}^{2}/{{|\chi }_{22}|}^{2}$ underlines the anisotropy ratio of THG emission ${I}_{x}^{(3\omega )}\left(\theta =0^{\circ}\right)/{I}_{y }^{(3\omega )}(\theta =90^{\circ})$ in the franckeite flakes, which almost remains as a constant of 1.51. Figure 4b plots the dependence of the THG emission power on the franckeite flake thickness for the incident linear polarization along the *x*-axis. The average pump power is kept at 1.1 mW corresponding to 8.65 GW/cm^2^ peak irradiance. It is found that the THG emission power gradually increases up to around 4 pW at the flake thickness of 97 nm with the conversion efficiency of 3.64 × 10^–9^, afterwards it exponentially decays. Such behavior of the thickness-dependent THG emission power can be explained in terms of the optical absorption dominance on the THG emission process as the flake thickness increases. Since franckeite is a semiconductor with a narrow band gap around 0.65 eV, there is strong optical absorption of franckeite crystal at the THG emission wavelength of ${\lambda }_{3}$ = 520 nm (2.38 eV). The strong optical absorption of franckeite especially at large flake thickness attenuates the generated THG signal to form the observed thickness-dependent exponential decay of THG emission power. Such thickness-dependent THG signal attenuation further enables us to extract the imaginary part of the refractive index (*k*_*3*_) at ${\lambda }_{3}$ = 520 nm for franckeite crystal by using the exponential fitting of THG emission power ${P}^{\left(3\omega \right)}\left(l\right)=A{l}^{2}\mathrm{exp}\left(-\frac{4\pi {k}_{3}l}{{\lambda }_{3}}\right)$, where *A* is a constant, *l* is the flake thickness. The exponential fitting of the measured THG emission power is plotted in Fig. 4b which gives *k*_*3*_ = 0.83. This value agrees with the previously reported *k*_*3*_ value for franckeite crystal at the wavelength of 520 nm, while the real part of the refractive index *n*_*3*_ is reported as around 3.2^30^. It is noted that there is some deviation between the measured THG power and the fitted data in Fig. 4b, especially the fitted values slightly overestimate the measured ones for franckeite thin flakes with thickness less than 70 nm. One potential reason for this inconsistency is that the mechanical exfoliation of franckeite thin flakes and the transfer process from tape to substrate might influence some top surface layers, resulting in a reduced THG conversion efficiency. Another possible explanation is the surface oxidation and contamination of franckeite thin flakes exposed to air, giving a lower THG emission power. In the future work, clean mechanical exfoliation in inert gas environment and surface encapsulation to protect the pristine franckeite crystals from the surface defects, oxidation and contamination can be utilized to solve such inconsistency issue.

Taken into account the values of *n*_*3*_, *k*_*3*_ and other experimental parameters of average pump power, laser pulse width, repetition rate, and spot size (${P}^{\left(\omega \right)}$ = 1.1 mW, $\tau$ = 90 fs, ${f}_{rep}$ = 80 MHz, and *W* = 1.5 µm) at the pump wavelength of 1560 nm, the magnitude of ${\chi }^{(3)}$ can be retrieved using the following equation^41^,

10 $${P}^{\left(3\omega \right)}\left(l\right)=\frac{9{\omega }^{2}{l}^{2}}{16\sqrt{{n}_{3}^{2}+{{k}_{3}}^{2}}{n}_{1}^{3}{\epsilon }_{0}^{2}{c}^{4}}{\left|{\chi }^{\left(3\right)}\right|}^{2}\frac{{{P}^{\left(\omega \right)}}^{3}}{{f}_{rep}^{2}{W}^{4}{\tau }^{2}{\left[\frac{\pi }{4\mathrm{ln}2}\right]}^{3}}\left(\frac{{e}^{-\frac{4\pi {k}_{3}l}{{\lambda }_{3}}}-2\mathrm{cos}\left(\Delta kl\right){e}^{-\frac{2\pi {k}_{3}l}{{\lambda }_{3}}}+1}{{l}^{2}\left(\frac{4{\pi }^{2}{{k}_{3}}^{2}}{{\lambda }_{3}^{2}}+{\Delta k}^{2}\right)}\right){e}^{-\frac{4\pi {k}_{3}l}{{\lambda }_{3}}}$$

where ${n}_{1}$ is the real part of the refractive index of franckeite crystal at the fundamental wavelength ${\lambda }_{1}$= 1560 nm and here ${n}_{1}$ = 4 is selected, and $\Delta k= \frac{6\pi }{{\lambda }_{1 }} ({n}_{1}- {n}_{3})$ is the phase mismatch between the fundamental beam and the forward propagating third-harmonic emission beam in the transmission microscope optical setup arrangement. The extracted magnitude of ${\chi }^{(3)}$ for franckeite crystal is 1.87 × 10^–19^ m^2^/V^2^. It is worth noting that several experimental parameters in Eq. (10), especially average pump power *P*^(ω)^, spot size *W*, and flake thickness *l* due to surface roughness, may affect the measured THG signal and propagate the error in determining the ${\chi }^{(3)}$ value. By considering the potential errors in average pump power *P*^(ω)^ = 1.1 ± 0.1 mW, spot size *W* = 1.5 ± 0.06 µm and flake thickness *l* ± 2 nm, it is found that the extracted ${\chi }^{(3)}$ value has the variation of less than ± 8%. Furthermore, while estimating the ${\chi }^{(3)}$ value from Eq. (10), the real part of the refractive index at 1560 nm is selected as *n*_1_ = 4, which is close to the reported value of franckeite in the near-infrared wavelength range with *n* = 4.1 at 777 nm^30^. By taking into account the uncertainty of the refractive index value selection, the *n*_1_ value is varied from 3 to 6 in the calculation which results in the ${\chi }^{(3)}$ value from 1.17 × 10^–19^ m^2^/V^2^ to 5.28 × 10^–19^ m^2^/V^2^. It shows that the variation of *n*_1_ does not induce an abrupt change in the ${\chi }^{(3)}$ value which remains in the same order of magnitude. The extracted ${\chi }^{(3)}$ value can be further justified by the previously reported ${\chi }^{(3)}$ value of layered franckeite as 5.1 × 10^–11^ esu (7.13 × 10^–19^ m^2^/V^2^) measured by the spatial self-phase modulation method with an ultrafast laser^23^, which is within the same order of magnitude. Additionally, in order to validate natural vdW heterostructures as an attractive alternative for advanced photonic and optoelectronic applications, the extracted ${\chi }^{(3)}$ value for natural vdW heterostructures is compared with the previously reported values for other nonlinear 2D material flakes such as black phosphorous (1.4 × 10^–19^ m^2^/V^2^), graphene (1.5 × 10^–19^ m^2^/V^2^), and MoS_2_ (2.9 × 10^–19^ m^2^/V^2^)^41,46,47^, showing that the ${\chi }^{(3)}$ value for franckeite crystal has the similar magnitude as other 2D materials.

## Discussion

In summary, we have demonstrated how the angle-resolved polarized Raman spectroscopy and the polarization-dependent THG emission can be utilized in probing the anisotropic natural vdW heterostructures with crystal structural deformation and identifying the direction of symmetry-broken periodic ripples induced by the lattice incommensurability. Both the highly anisotropic Raman response and THG emission pattern are observed from mechanically exfoliated franckeite thin flakes. We have discussed how the lattice incommensurability and in-plane spatial modulation of vdW forces between the consecutively stacked PbS-like and SnS_2_-like layers play a significant role in determining the anisotropic nature of nonlinear optical process in franckeite crystal. In order to further substantiate the experimental observations, the measured THG response is corroborated with the theoretical nonlinear susceptibility model to determine the relative magnitudes of the ${\chi }^{(3)}$ tensor elements and the value of ${\chi }^{(3)}$ for franckeite crystal. We have also discussed how the THG emission power from franckeite flakes is a cumulative effect of both nonlinear THG emission and linear optical absorption processes, which can be utilized for tailoring nonlinear light–matter interactions in multifunctional photonic integrated circuits. The results discussed here not only provide the comprehensive understanding of nonlinear optical processes in anisotropic natural vdW heterostructures, but also pave the way to the realization of integrated quantum photonics devices and circuits. Additionally, the electrically tunable THG signal modulation has been demonstrated^48,49^, where the order of magnitude of THG conversion efficiency in graphene is tuned by controlling the Fermi energy and the incident photon energy. Beyond graphene and transition metal dichalcogenides like MoS_2_ and WSe_2_, the emergence of anisotropic vdW materials has facilitated an additional degree of freedom to tailor the nonlinear optical interactions based on intrinsic structural anisotropy and crystal symmetry, which will be further harnessed to realize future design of vdW heterostructure-based anisotropic nonlinear optoelectronic devices, such as the electrically tunable broadband frequency converters for applications in optical communication, secured data transmission, encrypted signal processing, and quantum information processing.

## Methods

### Sample preparation

The glass substrates with 1 cm × 1 cm are treated with deionized water and isopropanol followed by ultra-sonication for 10 min to remove the undesirable residues from the surface. The processed glass substrates are used for transferring the franckeite thin flakes, which are mechanically exfoliated using Nitto tape (SPV 224) from bulk natural franckeite mineral (from San José mine, Oruro, Bolivia).

### Optical setup

For collecting the Raman spectra, the sample is excited with a 632.8 nm He–Ne laser source using a 40× objective lens (NA = 0.65) and the back-reflected light is collected with the same objective lens. The incident linear polarization of laser beam is controlled using a linear polarizer and a rotating half-wave plate in the excitation path. The collected light is routed to a spectrometer (Horiba, iHR 520) using a beam splitter. An appropriate edge filter (Semrock, LP02-633RE-25) is engaged in the collection path to reject the laser light. The collected signal is passed through a linear polarizer analyzer in the collection path to further resolve the parallel and perpendicular components of Raman spectra. For collecting the THG signal, the sample is pumped with a femtosecond laser source at the wavelength of 1560 nm (pulse width 90 fs, repetition rate 80 MHz) using a 40× objective lens (NA = 0.65). The transmission light is collected through a 100× objective lens (NA = 0.7). The pump laser light is filtered out by introducing a shortpass filter in the collection path. The transmitted THG signal is then routed towards a spectrometer and an imaging charge-coupled device (CCD) camera for the measurements.

## References

[1] Liu Y, Huang Y, der Duan XV (2019). Waals integration before and beyond two-dimensional materials. Nature.

[2] Geim AK, Van der Grigorieva IV (2013). Waals heterostructures. Nature.

[3] Jariwala D, Marks TJ, Hersam MC (2017). Mixed-dimensional van der Waals heterostructures. Nat. Mater..

[4] Withers F (2015). Light-emitting diodes by band-structure engineering in van der Waals heterostructures. Nat. Mater..

[5] Si M, Liao P-Y, Qiu G, Duan Y, Ye PD (2018). Ferroelectric field-effect transistors based on MoS_2_ and CuInP_2_S_6_ two-dimensional van der Waals heterostructure. ACS Nano.

[6] Massicotte M (2016). Picosecond photoresponse in van der Waals heterostructures. Nat. Nanotechnol..

[7] Sun X (2018). Tunable ultrafast nonlinear optical properties of graphene/MoS_2_ van der Waals heterostructures and their application in solid-state bulk lasers. ACS Nano.

[8] Li Z (2017). Q-switching of waveguide lasers based on graphene/WS_2_ van der Waals heterostructure. Photon. Res..

[9] Guo X (2020). Efficient all optical plasmonic modulators with atomically thin Van Der Waals heterostructures. Adv. Mater..

[10] Dasgupta A, Yang X, Gao J (2020). Nonlinear beam shaping with binary phase modulation on patterned WS_2_ monolayer. ACS Photon..

[11] Dasgupta A, Gao J, Yang X (2019). Atomically thin nonlinear transition metal dichalcogenide holograms. Nano Lett..

[12] Dankert A, Dash SP (2017). Electrical gate control of spin current in van der Waals heterostructures at room temperature. Nat. Commun..

[13] Wang Q (2018). Nonvolatile infrared memory in MoS2/PbS van der Waals heterostructures. Sci. Adv..

[14] Joel I (2019). Coherent control of a hybrid superconducting circuit made with graphene-based van der Waals heterostructures. Nat. Nanotechnol..

[15] Liu Y, Weiss NO, Duan X, Cheng H-C, Huang Y, der Duan XV (2016). Waals heterostructures and devices. Nat. Rev. Mater..

[16] Novoselov K, Mishchenko OA, Carvalho OA, Neto AC (2016). 2D materials and van der Waals heterostructures. Science.

[17] Frisenda R (2018). Recent progress in the assembly of nanodevices and van der Waals heterostructures by deterministic placement of 2D materials. Chem. Soc. Rev..

[18] Nutting D (2020). Heterostructures formed through abraded van der Waals materials. Nat. Commun..

[19] Wang X, Xia F (2015). Stacked 2D materials shed light. Nat. Mater..

[20] Zong X (2020). Black phosphorus-based van der Waals heterostructures for mid-infrared light-emission applications. Light Sci. Appl..

[21] Molina-Mendoza AJ (2017). Franckeite as a naturally occurring van der Waals heterostructure. Nat. Commun..

[22] Velický M (2017). Exfoliation of natural van der Waals heterostructures to a single unit cell thickness. Nat. Commun..

[23] Li J (2020). Nonlinear optical response in natural van der Waals heterostructures. Adv. Opt. Mater..

[24] Frisenda R (2020). Symmetry breakdown in franckeite: Spontaneous strain, rippling, and interlayer Moiré. Nano Lett..

[25] Shu Z (2020). Growth of ultrathin ternary teallite (PbSnS2) flakes for highly anisotropic optoelectronics. Matter.

[26] Moëlo Y (2008). Sulfosalt systematics: A review. Report of the sulfosalt sub-committee of the IMA Commission on Ore Mineralogy. Eur. J. Miner..

[27] Ray K (2017). Photoresponse of natural van der Waals heterostructures. ACS Nano.

[28] Burzurí E, Vera-Hidalgo M, Giovanelli E, Villalva J, Castellanos-Gomez A, Pérez EM (2018). Simultaneous assembly of van der Waals heterostructures into multiple nanodevices. Nanoscale.

[29] Gusmão R, Sofer Z, Luxa J, Pumera M (2018). Layered franckeite and teallite intrinsic heterostructures: Shear exfoliation and electrocatalysis. J. Mater. Chem. A.

[30] Gant P (2017). Optical contrast and refractive index of natural van der Waals heterostructure nanosheets of franckeite. Beilstein J. Nanotechnol..

[31] Autere A, Jussila H, Dai Y, Wang Y, Lipsanen H, Sun Z (2018). Nonlinear optics with 2D layered materials. Adv. Mater..

[32] Makovicky E, Petříček V, Dušek M, Topa D (2011). The crystal structure of franckeite, Pb_21.7_Sn_9.3_Fe_4.0_Sb_8.1_S5_6.9_. Am. Miner..

[33] Smith GD, Firth S, Clark RJ, Cardona M (2002). First-and second-order Raman spectra of galena (PbS). J. Appl. Phys..

[34] Chandrasekhar H, Humphreys R, Zwick U, Cardona M (1977). Infrared and Raman spectra of the IV-VI compounds SnS and SnSe. Phys. Rev. B.

[35] Li M (2017). Revealing anisotropy and thickness dependence of Raman spectra for SnS flakes. RSC Adv..

[36] Smith A, Meek P, Liang W (1977). Raman scattering studies of SnS_2_ and SnSe_2_. J. Phys. C..

[37] Vogt H, Chattopadhyay T, Stolz H (1983). Complete first-order Raman spectra of the pyrite structure compounds FeS_2_, MnS_2_ and SiP_2_. J. Phys. Chem. Solids.

[38] Parize R, Cossuetm T, Chaix-Pluchery O, Roussel H, Appert E, Consonni V (2017). In situ analysis of the crystallization process of Sb_2_S_3_ thin films by Raman scattering and X-ray diffraction. Mater. Des..

[39] Sereni, P., Musso, M., Knoll, P., Blaha, P., Schwarz K., & Schmidt, G. Polarization‐dependent raman characterization of stibnite. Presented at *AIP Conference Proceedings*, ***1267,*** 1131 (2010).

[40] Hart L, Dale S, Hoye S, Webb JL, Wolverson D (2016). Rhenium dichalcogenides: Layered semiconductors with two vertical orientations. Nano Lett..

[41] Youngblood N, Peng R, Nemilentsau A, Low T, Li M (2017). Layer-tunable third-harmonic generation in multilayer black phosphorus. ACS Photon..

[42] Dasgupta A, Gao J, Yang X (2020). Anisotropic third harmonic generation in layered germanium selenide. Laser Photon. Rev..

[43] Sar H, Gao J, Yang X (2020). In-plane anisotropic third-harmonic generation from germanium arsenide thin flakes. Sci. Rep..

[44] Boyd R (2020). Nonlinear Optics.

[45] Yang X-L, Xie S-W (1995). Expression of third-order effective nonlinear susceptibility for third-harmonic generation in crystals. Appl. Opt..

[46] Hong S-Y, Dadap JI, Petrone N, Yeh P-C, Hone J, Osgood RM (2013). Optical third-harmonic generation in graphene. Phys. Rev. X.

[47] Woodward R (2016). Characterization of the second-and third-order nonlinear optical susceptibilities of monolayer MoS2 using multiphoton microscopy. 2D Mater..

[48] Soavi G (2018). Broadband, electrically tunable third-harmonic generation in graphene. Nat. Nanotechnol..

[49] Jiang T (2018). Gate-tunable third-order nonlinear optical response of massless Dirac fermions in graphene. Nat. Photon..

